# *In vivo* effect of two first-line ART regimens on inflammatory mediators in male HIV patients

**DOI:** 10.1186/1476-511X-13-90

**Published:** 2014-05-29

**Authors:** Vasiliki D Papakonstantinou, Maria Chini, Nikos Mangafas, George M Stamatakis, Nickolaos Tsogas, Alexandros B Tsoupras, Katherina Psarra, Elizabeth Fragopoulou, Smaragdi Antonopoulou, Panagiotis Gargalianos, Constantinos A Demopoulos, Marios-C Lazanas

**Affiliations:** 1 Faculty of Chemistry, National & Kapodistrian University of Athens, Panepistimioupolis Zografou, 15771 Athens, Greece; 2 3rd Internal Medicine Department-Infectious Diseases Unit, Red Cross General Hospital, Athens, Greece; 3 Department of Immunology and Histocompatibility, Evangelismos Hospital, Athens, Greece; 4 Department of Science Nutrition-Dietetics, Harokopio University, Athens, Greece; 5 1st Internal Medicine Department-Infectious Diseases Unit, “G. Gennimatas” Hospital, Athens, Greece

**Keywords:** Platelet activating factor, Cytokines, Inflammation, Human immunodeficiency virus, Cardiovascular disease, Tenofovir-DF, Abacavir, Efavirenz

## Abstract

**Background:**

Persistent immune activation and inflammation are lying behind HIV-infection even in the setting of ART mediated viral suppression. The purpose of this study is to define the *in vivo* effect of two first-line ART regimens on certain inflammatory mediators in male HIV patients.

**Methods:**

Male, naive, HIV-infected volunteers were assigned either to tenofovir-DF/emtricitabine/efavirenz (Group_T) or abacavir/lamivudine/efavirenz (Group_A). Platelet Activating Factor (PAF) levels and metabolic enzymes together with HIV-implicated cytokines (IL-1beta, IL-6, IL-8, IL-10, IL-12p70, TNFa) and VEGF were determined for a 12-month period. Differences within each group were determined by non-parametric Friedman and Wilcoxon test, while the differences between the groups were checked by ANOVA repeated measures.

**Results:**

Both ART regimens present pronounced effect on inflammatory mediators, resulting in decreased PAF levels and Lipoprotein-associated phospholipase A2 (Lp-PLA2) activity for tenofovir-containing regimen and same as baseline PAF levels with a peak though at the 3^rd^ month as well as elevated Lp-PLA2 activity for abacavir-containing regimen.

**Conclusions:**

Studies regarding the effect of first-line ART regimens on inflammation may be beneficial in preventing chronic morbidities during HIV-treatment. From this point of view, the present study suggests an anti-inflammatory effect of tenofovir-containing ART, while the temporary increase of PAF levels in abacavir-containing ART may be the link between the reported cardiovascular risk and abacavir administration.

## Background

The progress of ART over the years has turned HIV-infection from lethal into a chronic disease. However, immune activation and inflammation are lying behind effectively treated patients causing various non-AIDS morbidities as cardiovascular or renal diseases and cancers [1]. Consequently chronic HIV-associated inflammation is now considered a significant factor for the above-mentioned conditions while elevated inflammatory and coagulation markers can predict higher morbidity and even mortality in HIV infected population [2-4].

During HIV-infection specific cells of the immune system become targets of the virus and secrete in response several inflammatory factors as cytokines and chemokines [5]. The effect of antiretrovirals on cytokines’ levels seems contradictory. Abacavir is thought to be associated with increased cardiovascular risk as it up-regulates pro-inflammatory cytokines and CRP [6,7]. Nevertheless, its use was not always associated with increased levels of inflammatory markers [8], showing a similar effect with tenofovir-DF [9]. In addition both antiretrovirals had no effect on coronary endothelial cell gene transcription and protein expression of cytokines, supporting that the increased cardiovascular risk is probably not through the direct endothelial activation pathways [10]. The inconsistent effect of ART on cytokines’ levels maybe indicates another inflammatory mediator to be responsible for the increased cardiovascular risk associated with abacavir use.

Platelet Activating Factor (PAF) is one of the most potent inflammatory factors and a significant signaling molecule of the immune system, considered as a primitive and universal cellular mediator [11,12] that participates in both physiological and pathological processes [13,14]. Concerning PAF biosynthesis, there are two distinctive pathways named *de novo,* responsible for the constant PAF biosynthesis*,* and remodeling*,* activated as a direct response to acute inflammatory processes [15], with key-enzymes being dithiothreitol-insensitive PAF-cholinephosphotransferase (PAF-CPT, EC 2.7.8.16) and lyso-PAF:acetyl-CoA acetyltransferase (lyso-PAF-AT, EC 2.3.1.67) [16,17], respectively. PAF catabolism is moderated by PAF-specific acetylhydrolase (PAF-AH, EC 3.1.1.47), and its plasma isoform, Lipoprotein-associated phospholipase A2 (Lp-PLA2) [18].

PAF is thought to be implicated in the progression of HIV-infection as studies have revealed that HIV-infected monocytes overexpress PAF through Tat protein [19,20]. It was found that altered host cells and their sub-products as Tat protein induce PAF biosynthesis via cytokines, as Tumor Necrosis Factor-alpha (TNFα), and growth factors, as Vascular Endothelial Growth Factor (VEGF) [21,22]. The produced PAF, as a secondary mediator, participates in several processes like monocyte cell recruitment and increased vascular permeability, which can lead to various morbidities [2,23,24]. A great variety of antiretrovirals, backbone and HAART regimens exhibit *in vitro* inhibitory effect against PAF activity [25] while many studies have underlined the need for combined antiretroviral and anti-PAF action in drugs like piperazine and its developed derivatives [26-28]. Patients with early or asymptomatic HIV-infection before ART initiation have shown increased activity of PAF biosynthetic enzymes and Lp-PLA2 indicating a persistent inflammatory condition [29]. It has also been proposed that Lp-PLA2 could play the role of a sensitive marker as its increased levels in HIV and AIDS patients may be a physiological response protecting the host against PAF or other oxidized phospholipids [30]. The implication of PAF in HIV-infection is further supported by the beneficial effect of PAF antagonists administration in animal models for HIV-1 encephalitis [31,32] as well by the improvement of neuropsychological test scores in HIV-patients under lexipafant, the first PAF antagonist used in HIV-associated cognitive impairment [33].

The scope of this paper is to determine for the first time the *in vivo* effect of two first line ART regimens on PAF levels in HIV naive patients along with several already established inflammatory biomarkers implicated in HIV-infection. The hypothesis states that tenofovir-DF/emtricitabine/efavirenz would down regulate PAF levels in contrast to abacavir/lamivudine/efavirenz based on preliminary data concerning the effect of the above regimens on PAF enzymes [34,35].

## Methods

### Study design

Study enrollment begun after obtaining approval from the scientific board of the Red Cross General Hospital of Athens in Greece and all volunteer patients have signed the informed consent according to the Declaration of Helsinki. The volunteers (n = 18) were recruited from the 3^rd^ Internal Medicine Department-Infectious Diseases Unit, Red Cross General Hospital, Athens, Greece. All participants were male, treatment naïve, and asymptomatic HIV-infected patients as determined by the presence of antibodies against HIV measured by enzyme-linked immunosorbent assay (ELISA) and confirmed by Western blot. All patients were at CDC A2 clinical stage and fulfilled the criteria for ART initiation according to the European [36] and International guidelines [37]. The patients were assigned in 2 groups at the discretion of the clinicians. Group_T (n = 8, mean age = 46 ± 10 years, 50% smokers) received co-formulated tenofovir-DF/emtricitabine with efavirenz and Group_A (n = 10, mean age = 35 ± 11 years, 70% smokers) received co-formulated abacavir/lamivudine along with efavirenz. The exclusion criteria include inflammatory diseases (periodontal or autoimmune disease, other diseases (diabetes, hypertension), allergies or any additional medication that may affect PAF levels. The study lasted for 12 months and the blood samples were collected before (baseline, defined as 0 months) and after 1, 3, 6, 9 and 12 months of treatment initiation.

### Determination of PAF levels

The isolation and purification of PAF was according to the method of Demopoulos et al. [38]. Briefly, 10 mL of blood were collected from each human subject and poured immediately into 40 mL of absolute ethanol. The mixture was stirred and centrifuged at 300 × g for 10 min at room temperature. The supernatant and the pellet were extracted separately according to the Bligh and Dyer method [39] and the chloroform phase in each case was stored at -20°C. The supernatant chloroform extract contains PAF molecules that are loosely bounded to plasma proteins and lipoproteins, named as free PAF, while the pellet extract contains PAF molecules strongly bounded to cellular structures, named as bound PAF. The above extracts were purified on silicic acid column chromatography that was eluted with 45 mL of methanol/water (1:1.5, v/v), followed by 50 mL of methanol/water (2:1, v/v). The initial 45 mL (containing the bulk of proteinaceous and other non-lipid impurities) were discarded while the PAF containing eluents were further purified by HPLC (Hewlett-Packard series 1100) on a cation-exchange column. The solvent system consisted of an isocratic elution of acetonitrile/methanol/water (61:31:8, v/v/v) slightly modified from the one described in the original paper [38]. The eluted substances were detected using UV detection at 208 nm. The examined samples were dissolved in BSA 1.25% in saline and its PAF levels were measured by the aggregatory activity (by a 400 VS aggregometer Chrono-Log, USA) towards washed rabbit platelets. PAF levels are expressed as fmol/mL of blood.

### Isolation of plasma, platelets and leukocytes

The isolations of plasma, platelets (HPs) and leukocytes (HLs) were carried out as previously described [40]. Briefly, human blood was collected from an antecubital vein and distributed into a polyethylene tube containing anticoagulant in a ratio of blood/anticoagulant: 9/1, in a total volume of 10 mL. Each sample underwent multiple centrifugations in order to isolate plasma, platelets and leukocytes.

### Enzymatic assays

Dithiothreitol-insensitive PAF-CPT and lyso-PAF-AT activity assays were performed on the homogenates of leukocytes and platelets. PAF-AH activity assays were performed on the homogenates of leukocytes and platelets as well as Lp-PLA2 activity assays on plasma as previously described [40]. The enzyme activity is expressed as pmol/min/mg of total protein or pmol/min/μL of plasma.

### Biochemical markers and immunological analysis

Clinical biochemical markers were measured by a Siemens Dimension RxL automatic analyzer. CD4 cell counts were defined using Tetra One System on the EPICS XL flow cytometer, while viral load was determined using the Versant HIV-1 RNA 3.0 assay. Plasma levels of IL-1β, IL-6, IL-8, IL-10, IL-12p70, TNFα and VEGF were determined on a BD FACS Canto II Flow cytometer using CBA flex set cytokine kit and data analysis was performed by FCAP Array DIVA software. Cytokines are expressed as pg/mL of plasma.

### Statistical analysis

Normal distribution was checked by P-P graphs using Shapiro-Wilk criterion. The results are expressed as median values and interquartile range (25^th^-75^th^). Differences within each group during the overall 12-month treatment were determined by non-parametric Friedman (displayed as p_time_) analysis. Wilcoxon test was used to compare the difference of specific time point with the baseline values within each group (displayed as p_specific-time-point_). After ranking the values, the differences between the groups were checked by repeated measures ANOVA and are displayed as P_intervention_ (P_int_) for the overall and P_specific-time-point_ for each time point in particular. Viral load changes are reported in a logarithmic scale for convenience and not in order to normalize the value. Statistical significance was considered as p < 0.05.

## Results

### Anthropometric and biochemical characteristics

Anthropometric and biochemical characteristics of patients are shown in Additional file 1, presenting no difference between the two groups (P_int._s > 0.05).

In both groups, viral load is progressively reduced during the study period (p_time_A/T_ < 0.001), while CD4 cell counts are gradually increased (p_time_A/T_ < 0.001) even from the 1^st^ month of treatment. White blood cell count remains stable in both groups (p_time_T_ = 0.205, p_time_A_ = 0.091), belonging to the minimum level of the normal range.

Despite the variations in the lipid profile of each group, the values remain within the normal range. Particularly, in both groups total cholesterol (p_time_T_ = 0.007, p_time_A_ = 0.001), LDL (p_time_T_ = 0.028, p_time_A_ = 0.007) and HDL (p_time_T_ = 0.008, p_time_A_ = 0.002) are increased while triglycerides remain stable (p_time_T_ = 0.170) in Group_T and are increased in Group_A (p_time_A_ = 0.001).

### Effect of ART regimens on PAF levels

**Figure 1:**
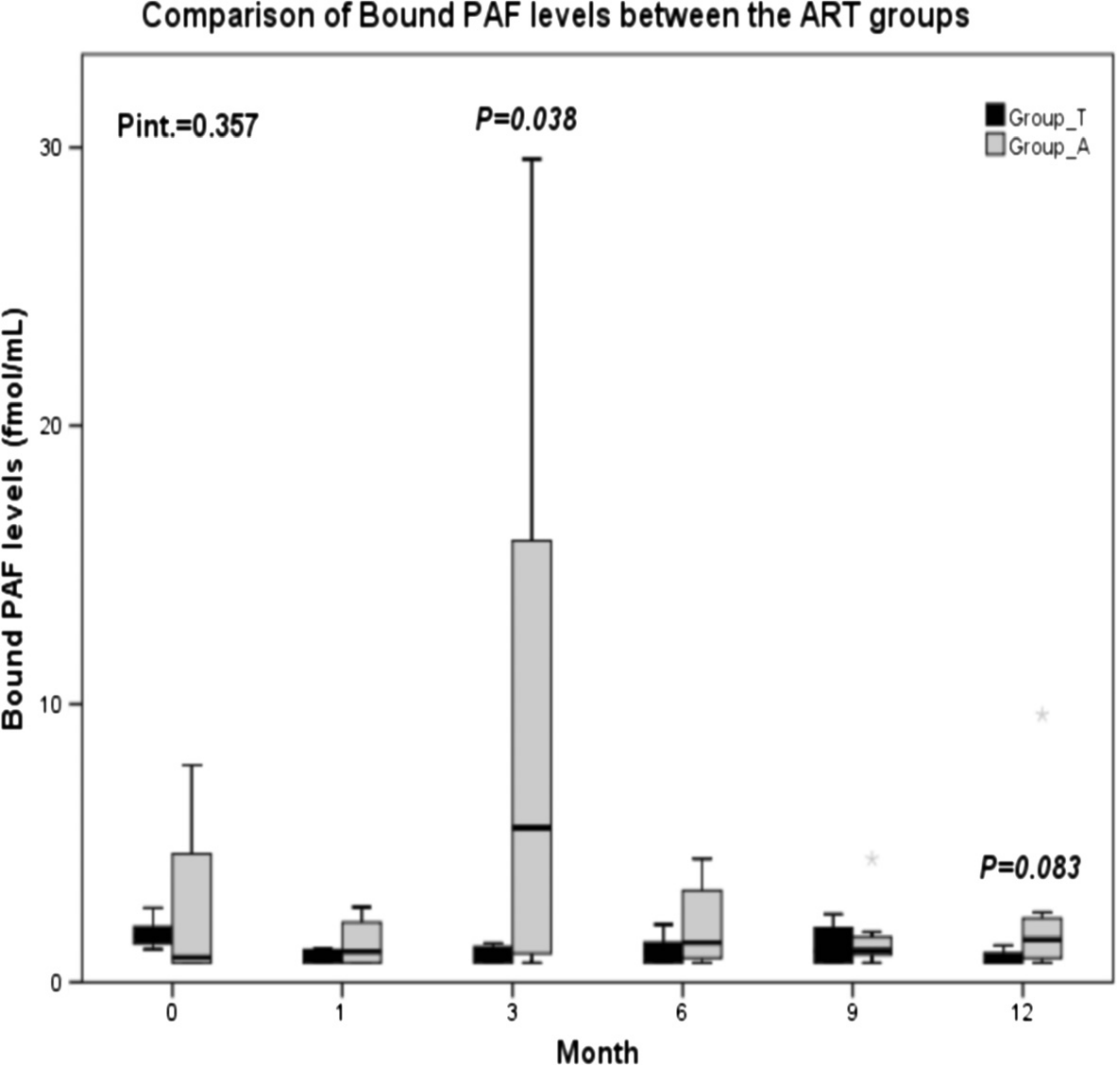
**Comparison of Bound PAF levels between the ART groups, Group_T: tenofovir-DF/emtricitabine/efavirenz, Group_A: abacavir/lamivudine/efavirenz, Data are expressed as median values and interquartile range (25**^**th**^**-75**^**th**^**).** Pint. displays the overall difference between the two groups. P displays the difference at a particular time point of the study occurred from the comparison of the two groups.

Bound PAF levels in Group_T are differentiated through the 12-month period (p_time_T_ = 0.010) and especially they are decreased at the 1^st^, 3^rd^, 6^th^ and 12^th^ month (p_1_ = 0.008, p_3_ = 0.016, p_6_ = 0.039 and p_12_ = 0.008, respectively). Bound PAF levels in Group_A are also differentiated during the study period (p_time_A_ = 0.028), with a single increase at the 3^rd^ month (p_3_ = 0.016), (Table 1). There is no overall differentiation between the groups (P_int_ = 0.357), however a differentiation is achieved at the 3^rd^ month (p_3_A-T_ = 0.038) and a borderline one at the 12^th^ month (p_12_A-T_ = 0.083); (Figure 1).

**Table 1:** PAF levels and metabolic enzymes of ART groups

**Parameter**	**Groups**	**Baseline/0 month**	**1**^**st **^**month**	**3**^**rd **^**month**	**6**^**th **^**month**	**9**^**th **^**month**	**12**^**th **^**month**	**p**_**time**_	**P**_**int.**_
**Bound PAF (fmol/mL)**	T	1.66	1.11*	0.90*	1.08*	1.12	0.74*	**0.010**	0.357
		(1.35-2.02)	(0.70-1.18)	(0.70-1.30)	(0.70-1.54)	(0.70-2.11)	(0.70-1.18)		
	A	0.90	1.10	5.54*	1.42	1.14	1.53	**0.028**	**P**_**3 **_**= 0.038**
		(0.70-6.20)	(0.70-2.41)	(0.97-17.29)	(0.78-3.85)	(0.93-1.71)	(0.78-2.39)		**P**_**12 **_**= 0.083**
**PAF-CPT in HLs (pmol/min/mg)**	T	546.50	336.00*	191.50*	232.00*	338.50*	288.50*	**0.009**	0.304
		(416.50-730.25)	(182.50-451.75)	(114.50-518.00)	(147.25-461.25)	(231.25 -381.25)	(236.50-341.75)		
	A	344.50	257.00	438.00	344.00	495.00	445.00*	0.085	
		(215.25-787.75)	(221.75-515.25)	(183.00-535.50)	(173.50-795.50)	(252.50-755.00)	(330.00-920.00)		
**Lyso-PAF-AT in HLs (pmol/min/mg)**	T	22.95	12.40	12.20	8.40*	13.85*	16.40*	**0.002**	0.818
		(11.90-26.65)	(10.75-33.27)	(8.02-28.07)	(2.22-12.92)	(8.47-17.60)	(9.77-19.75)		
	A	9.15	10.75	20.60*	10.55	13.50	12.50	0.141	
		(6.12-19.82)	(5.25-22.20)	(7.42-35.75)	(6.85-27.27)	(8.00-27.75)	(6.50-24.25)		
**PAF-AH in HLs (pmol/min/mg)**	T	69.17	55.68	70.71	54.18*	46.32*	45.81*	**0.002**	0.732
		(42.94-106.97)	(32.34-80.52)	(37.47-114.14)	(33.09-65.45)	(28.23-62.67)	(25.79-51.56)		
	A	50.54	52.68	64.03*	61.30	57.05	52.24	0.180	
		(46.57-58.78)	(34.37-72.78)	(50.80-92.62)	(32.55-75.06)	(36.49-66.05)	(40.54-61.08)		
**Lp-PLA2 (pmol/min/μL)**	T	29.35	26.15	34.20	29.10	26.40*	27.15*	**0.003**	**0.017**
		(24.40-34.12)	(23.70-35.37)	(30.00-35.12)	(26.15-38.47)	(22.44-29.35)	(20.79-28.50)		
	A	33.66	32.63	33.59	33.67	37.55*	33.33	**0.002**	**P**_**3 **_**= 0.038**
		(30.30-35.89)	(27.20-36.59)	(29.32-38.15)	(27.61-35.48)	(35.67-42.43)	(31.63-40.90)		**P**_**12 **_**= 0.083**

Group_T: tenofovir-DF/emtricitabine/ efavirenz and Group_A: abacavir/lamivudine/ efavirenz. All the results are expressed as median values and interquartile range (25^th^-75^th^). HLs: Human Leukocytes. p_time_ displays the difference within the group during the overall 12-month treatment.

*: displays the significant difference of each time point with the baseline value (**p < 0.05**).

P_int_ displays the difference between the two groups during the overall 12-month treatment.

P_specific-time-point_ displays the significant difference between the two groups at a specific time point.

Regarding Free PAF levels, in Group_T they are borderline differentiated through the study period (p_time_T_ = 0.068) while in Group_A they remain stable throughout the treatment period (p_time_A_ = 0.719), with no differentiation at any time point for both groups nor between them (P_int_ = 0.917), (Additional file 2).

### Effect of ART regimens on PAF basic biosynthetic enzymes (PAF-CPT, lyso-PAF-AT) in leukocytes and platelets

As far as it concerns PAF biosynthesis in leukocytes, the specific activity of PAF-CPT in Group_T is differentiated during the 12-month period (p_time_T_ = 0.009) with gradually reductions from the 1^st^ to the 12^th^ month (p_1_ = 0.016, p_3_ = 0.008, p_6_ = 0.047, p_9_ = 0.008 and p_12_ = 0.008, respectively). In contrast, in Group_A it is borderline differentiated through the study period (p_time_A_ = 0.085), achieving however an increase, at the 12^th^ month (p_12_ = 0.006). Although the comparison of the two groups presents no overall differentiation (P_int_ = 0.304), there is a significant one at the 12^th^ month (p_12_A-T_ = 0.016). Regarding the specific activity of lyso-PAF-AT, in Group_T it is differentiated during the 12-month treatment (p_time_T_ = 0.002) with significant decreases from the 6^th^ to the 12^th^ month (p_6_ = 0.008, p_9_ = 0.008, p_12_ = 0.008, respectively). On the contrary, in Group_A, no difference is observed throughout the study period (p_time_A_ = 0.141), however, an increase is detected at the 3^rd^ month (p_3_ = 0.037); (Table 1).

Regarding PAF biosynthesis in platelets, in Group_T the specific activity of PAF-CPT remains stable through time (p_time_T_ = 0.343) with a reduction, however, at the 12^th^ month (p_12_ = 0.047). Besides, the specific activity of lyso-PAF-AT in Group_T remains stable through the 12-month period (p_time_T_ = 0.416), decreasing though at the 9^th^ month (p_9_ = 0.039), while in Group_A it is differentiated during the study period (p_time_A_ = 0.041) with a significant peak at the 3^rd^ month of treatment (p_3_ = 0.010); (Additional file 2).

### Effect of ART regimens on both catabolic isoforms of PAF-acetylhydrolase: Lp-PLA2 in plasma and PAF-AH in blood cells

**Figure 2:**
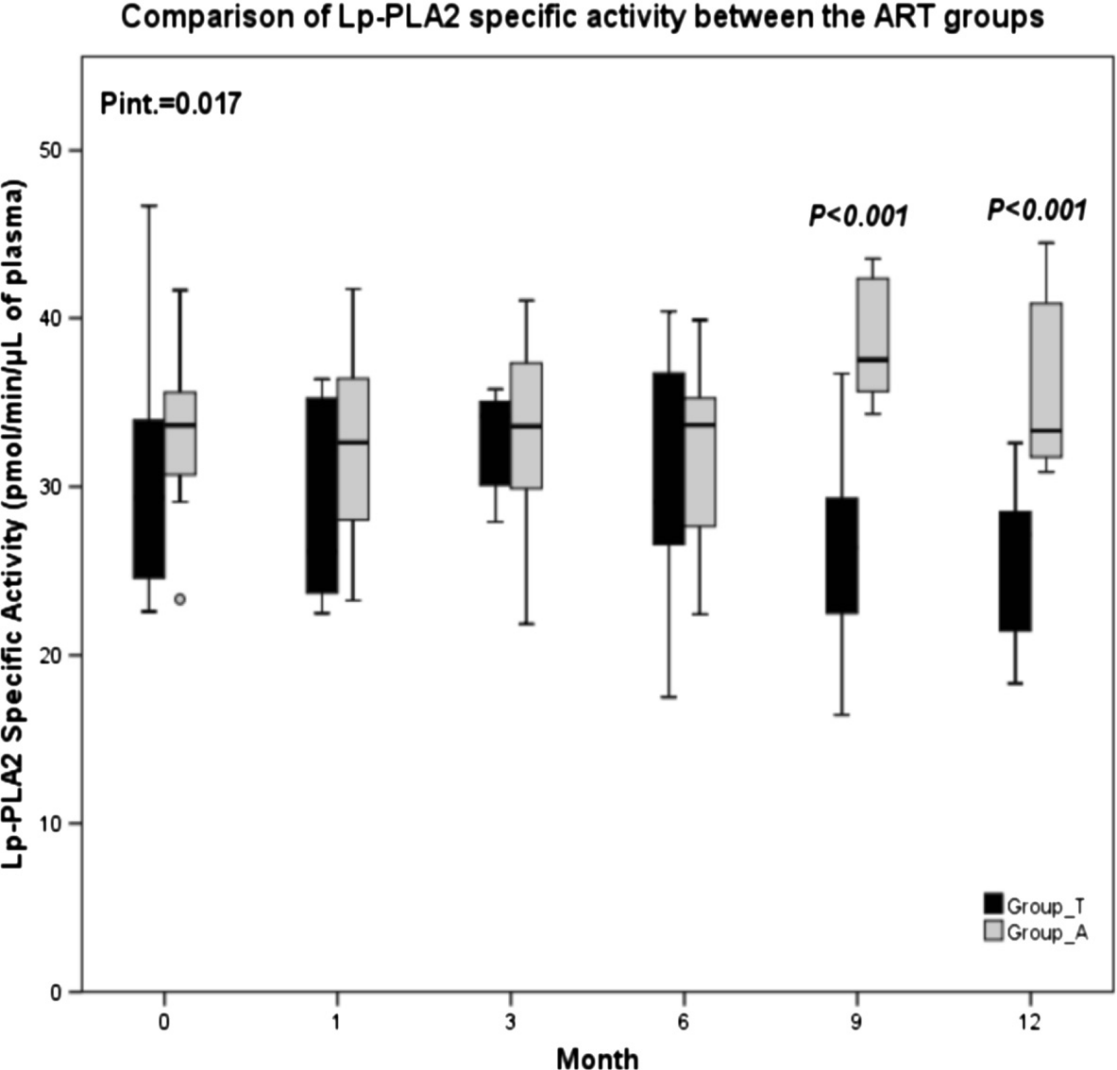
**Comparison of Lp-PLA2 specific activity between the ART groups, Group_T: tenofovir-DF/emtricitabine/efavirenz, Group_A: abacavir/lamivudine/efavirenz, Data are expressed as median values and interquartile range (25**^**th**^**-75**^**th**^**).** Pint. displays the overall difference between the two groups. P displays the difference at a particular time point of the study occurred from the comparison of the two groups.

The specific activity of Lp-PLA2 in plasma presents significant variations throughout the study period in both ART groups (p_time_T_ = 0.003 and p_time_A_ = 0.002); (Figure 2). Specifically, in Group_T it is decreased at the 9^th^ and 12^th^ month (p_9,12_ = 0.008), while in Group_A, it is increased at the 9^th^ month (p_9_ = 0.002), (Additional file 2).

Regarding the specific activity of PAF-AH in leukocytes, in Group_T it is differentiated through the 12-month period (p_time_T_ = 0.002), with significant decreases from the 6^th^ to the 12^th^ month (p_6_ = 0.031, p_9_ = 0.016 and p_12_ = 0.008, respectively). In contrast, in Group_A, the specific activity of PAF-AH is not differentiated during the study period, achieving however a significant peak at the 3^rd^ month (p_3_ = 0.049), (Table 1).

Concerning the specific activity of PAF-AH in platelets, only in Group_T it is differentiated through the 12-month period (p_time_T_ = 0.023), with significant reductions from the 6^th^ to the 12^th^ month (p_6_ = 0.016, p_9_ = 0.008 and p_12_ = 0.008, respectively), (Additional file 2).

### In vivo effect of ART regimens on cytokines’ levels

Cytokines’ levels remain stable through the study period in both groups (p’s_time_ > 0.05), although a single increase of TNFα levels in Group_A (p_1_ = 0.027) is observed at the 1^st^ month of treatment, (Additional file 3).

## Discussion

Over the last 18 years, ART succeed in raising CD4 cell counts and suppressing viral load which has remarkably improved the course of HIV-disease, with longer survival and improved quality of life [2]. At the same time, it has led to the appearance of previously unrecognized chronic complications, such as ischemic cardiovascular events, cancer and nephropathy [41]. It is not clear yet whether these manifestations are related to the ART administration or the virus itself. Even patients under antiretroviral therapy with undetectable viral load present long-term immune activation and chronic inflammation [1,2]. Therefore the blocking of the above mechanisms could be a parallel therapeutic approach. Although it has been suggested that persistent inflammation is implicated in chronic non-HIV related manifestations, it is not clear yet which inflammatory mediators could be used in clinical practice in order to reduce HIV morbidity and mortality.

Our team has already shown that tenofovir-DF/emtricitabine/efavirenz has great *in vitro* inhibitory effect against PAF [25] and decreases PAF biosynthesis during a 6-month treatment period [34] while abacavir/lamivudine/efavirenz has limited *in vitro* inhibitory effect [25] against PAF and increases at the 3^rd^ month lyso-PAF-AT, which is implicated in acute inflammatory processes [35]. The present study expands the previous data by providing the effect of these ART regimens on PAF levels during a 12-month period and the follow-up of PAF metabolic enzymes’ activity at the 9^th^ and 12^th^ month along with the levels of selected HIV-implicated cytokines (IL-1β, IL-6, IL-8, IL-10, IL-12p70, TNFα and VEGF).

In the present study, PAF levels in HIV patients are measured for the first time, resulting surprisingly to lower values than the average population although the activity of PAF biosynthetic enzymes is much higher [42]. One may suggest that a possible explanation may be the increased activity of PAF catabolic enzyme, namely PAF-AH for the cellular isoform and Lp-PLA2 for the plasma isoform, acting in order to counterbalance PAF levels in blood. In addition, another possible explanation for PAF decreased levels is the lower white blood cell counts in HIV patients compared to healthy population. The comparison of PAF levels between the two groups reveals a significant increase in Group_A at the 3^rd^ month. This differentiation is probably attributed to the peak of lyso-PAF-AT activity at the 3^rd^ month in Group_A in parallel with the continual decrease of PAF biosynthesis in Group_T. There is also a trend of difference at PAF levels noted at the 12^th^ month, which may be credited to the significant difference at the 12^th^ month of PAF-CPT as well to the opposite effect of the two regimens on both biosynthetic enzymes. The above results of higher PAF levels and metabolic enzymes’ activity, seen at certain time points, support a possible direct effect of abacavir than an indirect effect of immune reconstitution as it occurs from the white blood cell count.

In this study the activity of Lp-PLA2, a well-known cardiovascular risk marker and sensitive to PAF levels [43], is higher than the average population [42]. Increased PAF levels seem to increase Lp-PLA2 activity in order to adjust PAF levels in blood and this may be the reason of the significant overall differentiation of Lp-PLA2 activity between the two groups spotted particularly at the 9^th^ and the 12^th^ month. Moreover, in Group_T the decrease of Lp-PLA2 at the 9^th^ and the 12^th^ month comes in accordance with the decrease in PAF levels during the 12-month period. In Group_A the increase of PAF levels at the 3^rd^ month may be the reason of Lp-PLA2 raise at the 9^th^ month or this raise possibly reflects a direct effect of abacavir/lamivudine/efavirenz on this enzyme.

The inflammatory cytokines IL-1β, IL-6, IL-10, IL12p70 and TNFα are implicated both at the pathogenesis and the morbidities of HIV infection. In the present study, cytokines’ levels are higher than healthy population indicating the immune state of these HIV patients [44]. Cytokines’ levels remain stable between and within ART groups during the study period apart from an increase of TNFα levels at the 1^st^ month in Group_A. The elevated TNFα levels may assist to the increased PAF levels at the 3^rd^ month, knowing that TNFα induces PAF biosynthesis and *vice versa*[45,46].

## Conclusion

In conclusion the present study points out the effect of ART regimens to the persistent inflammatory state observed in the setting of suppressive antiretroviral treatment. The tested regimens caused an opposite outcome on PAF levels and on Lp-PLA2 activity. Since PAF is a potent inflammatory factor, associated with pro-atherosclerotic complications, the above results indicate a potent anti-inflammatory role for tenofovir based regimen while suggest PAF as the missing link between the abacavir based regiment and the reported cardiovascular risk.

### Study limitations

The main limitation of this research study is the number of the samples. The reason is the chosen methods for PAF which are quite strenuous and time consuming but also more sensitive and trustful than other rapid methods. Female patients have not been used in the study as the menstrual cycle affects the metabolism of PAF.

## Competing interests

The authors declare no competing Interests. The research has been funded from the Hellenic Society for the Research, Study and Education in Infectious Diseases.

## Authors’ contributions

V.D.P is responsible for the authorship, the experiments and the data analysis. G.S and A.B.T participated at the experiments and the data analysis. M.C, N.M and N.T designed the selection of patient samples, provided the clinical data for analysis and contributed to the interpretation of data, revising drafts critically for important intellectual content and final approval of the version to be published. E.F conducted the data analysis and reviewed the manuscript. S.A critically reviewed the manuscript. C.A.D and M.C.L conceived and supervised the whole study. All authors read and approved the final manuscript.

## Supplementary Material

Additional file 1 Anthropometric and biochemical characteristics of ART groups. Click here for file

Additional file 2 Free PAF levels and metabolic enzymes in platelets of ART groups. Click here for file

Additional file 3 Cytokines and VEGF levels in HIV-infected patients. Click here for file
