# Supplementary material for: In vivo effect of two first-line ART regimens on inflammatory mediators in male HIV patients
Source: Lipids Health Dis. 2014 May 29;13:90. doi: 10.1186/1476-511X-13-90 (PMC4055908; doi:10.1186/1476-511X-13-90)
Supplement: Additional file 1 — Anthropometric and biochemical characteristics of ART groups. [file 1476-511X-13-90-S1.pdf]

Additional File 1: Anthropometric and biochemical characteristics of ART groups

**Characteristics**	**Groups**	**Baseline / 0 month**	**1st month**	**3rd month**	**6th month**	**9th month**	**12th month**	**p_{time}**	**$P_{int.}$**
**CD4 (cells/µL)**	T	273.0 (232.0 - 362.0)	348.0* (319.0 – 412.0)	375.0* (315.0 – 422.0)	411.5* (279.5 – 470.25)	479.0* (390.5 – 534.25)	439.0* (329.0-478.7)	**<0.001**	0.349
	A	313.5 (246.2 - 323.0)	372.0* (350.7 – 424.0)	473.5* (404.2 – 505.7)	467.5* (387.7 – 515.5)	526.5* (361.5 – 614.0)	526.5* (379.2-626.7)	**<0.001**	
**Viral load (log copies/mL)**	T	4.3 (4.0 – 4.7)	2.4* (2.2 – 2.7)	ND*	ND*	ND*	ND*	**<0.001**	0.065
	A	4.4 (3.8 – 5.1)	2.4* (1.9 – 2.6)	2.0* (2.0 – 2.0)	ND*	ND*	ND*	**<0.001**	
**BMI (Kg/m^2)**	T	27.4 (24.4 - 32.6)	27.5 (24.5 – 31.4)	27.7 (24.4 – 30.9)	28.1 (24.8 – 30.1)	28.1 (24.7 – 29.9)	28.0 (24.1 - 30.6)	0.947	0.413
	A	24.2 (23.4 - 27.6)	24.4 (23.6 - 27.8)	24.1 (23.9 - 28.7)	24.6 (23.6 - 29.2)	24.8 (23.2 - 28.5)	24.7 (24.0 - 28.4)	0.333	
**Total Cholesterol (mg/dL)**	T	169.5 (157.2 - 193.7)	188.0* (179.0 - 219.7)	209.0* (179.5 – 255.0)	198.0* (189.2 – 240.0)	199.5* (183.5 – 221.5)	197.0 * (183.2 – 245.0)	**0.007**	0.408
	A	156.0 (136.8 - 179.5)	185.5* (169.7 - 263.7)	194.5* (170.0 – 241.2)	197.0* (171.0 – 243.7)	213.5* (176.0 – 244.2)	202.5 * (184.5 – 240.2)	**0.002**	
**HDL (mg/dL)**	T	34.0 (28.0 - 40.5)	42.0 (37.0 - 47.0)	46.0* (32.2 - 50.7)	46.5* (33.7 – 53.7)	44.5* (32.7 – 51.0)	40.5* (33.0 - 48.7)	**0.008**	0.392
	A	34.5 (29.0 - 38.8)	41.5* (32.7 - 48.5)	42.0* (33.5 - 50.0)	43.5* (40.7 – 48.7)	42.5* (37.7 – 47.2)	38.0 * (37.0 - 50.7)	**0.002**	
**LDL (mg/dL)**	T	120.0 (105.2 - 136.2)	133.0 (115.0 – 152.0)	137.0* (115.7 – 188.2)	142.5* (114.0 – 150.0)	141.5* (121.5 – 152.7)	147.7* (117.0 - 176.2)	**0.028**	0.417
	A	98.0 (80.0 - 125.0)	116.0* (85.5 – 145.2)	107.0* (96.7 – 155.5)	113.5* (105.2 – 142.7)	121.0 (91.7 – 145.7)	117.5* (98.7 - 146.0)	**0.007**	

**Triglycerides (mg/dL)**	T	104.5 (63.5 - 133.7)	108.5 (69.0 – 172.5)	114.0 (83.7 – 146.7)	101.5 (79.7 – 131.5)	112.5 (68.2 – 148.2)	105.5 (79.2 – 158.0)	0.170	0.378
	A	77.5 (62.8 - 213.3)	117.0* (93.0 – 387.7)	122.0* (91.7 – 251.5)	122.5* (89.7 – 238.2)	147.5* (92.5 – 548.5)	129.0* (111.7- 354.0)	**0.001**	
**Glucose (mg/dL)**	T	92.0 (87.0 - 111.0)	98.5 (94.2 – 126.2)	91.5 (87.2 – 114.5)	104.5 (92.0 – 110.2)	100.0 (86.5 – 107.7)	100.0 (88.2-103.2)	0.167	0.384
	A	86.0 (76.7 - 100.7)	95.0 (91.6 – 100.0)	95.0* (90.7 – 103.2)	95.0* (90.7 – 103.2)	97.5* (86.2 – 104.0)	97.0 (88.7-101.0)	0.143	
**White Blood Cell Count (10^3/μL)**	T	5.0 (4.2 – 5.4)	5.1 (4.1 – 6.3)	5.4 (4.0 – 7.2)	5.6 (5.1 – 6.7)	5.5 (4.5 – 7.5)	5.9* (5.1 – 7.0)	0.205	0.352
	A	5.3 (4.6 – 6.3)	5.1 (4.2 – 6.9)	5.2 (4.4 – 6.1)	6.3 (4.7 – 7.2)	6.0 (4.8 – 6.8)	5.8 (5.3 – 8.0)	0.091	

Group_T: tenofovir-DF/emtricitabine/ efavirenz, Group_A: abacavir/lamivudine/ efavirenz. All the results are expressed as median values and interquartile range (25th-75th).

p_{time} and $P_{int.}$ display the difference within each groups or between the two groups during the overall 12-month treatment, respectively

*: displays the significant difference of each time point with the baseline value ($p<0.05$).
