# Supplementary material for: In vivo effect of two first-line ART regimens on inflammatory mediators in male HIV patients
Source: Lipids Health Dis. 2014 May 29;13:90. doi: 10.1186/1476-511X-13-90 (PMC4055908; doi:10.1186/1476-511X-13-90)
Supplement: Additional file 2 — Free PAF levels and metabolic enzymes in platelets of ART groups. [file 1476-511X-13-90-S2.pdf]

Additional File 2: Free PAF levels and metabolic enzymes in platelets of ART groups

Parameter	Groups	Baseline / 0 month	1st month	3rd month	6th month	9th month	12th month	p_{time}	$P_{int.}$
**Free PAF (fmol/mL)**	T	0.99 (0.63-2.64)	0.64 (0.62-0.74)	0.62 (0.62-0.88)	1.50 (0.65-2.73)	0.62 (0.62-1.83)	0.62 (0.62-0.69)	0.068	0.917
	A	0.61 (0.25-0.75)	0.70 (0.37-1.51)	0.77 (0.37-1.88)	1.48 (0.40-2.02)	1.15 (0.25-2.58)	0.81 (0.25-1.84)	0.719	
**PAF-CPT in HPs (pmol/min/mg)**	T	213.00 (94.00-256.00)	145.50 (73.75-187.25)	142.50 (74.75-288.00)	170.00 (119.50-208.75)	152.00 (136.75-197.25)	151.50* (112.25-177.50)	0.343	0.438
	A	143.00 (68.25-205.75)	117.00 (54.50-173.50)	142.50 (66.50-166.25)	139.50 (71.00-184.50)	150.00 (77.50-182.50)	135.00 (67.50-185.00)	0.757	
**Lyso-PAF-AT in HPs (pmol/min/mg)**	T	38.50 (13.00-46.00)	25.50 (10.00-45.50)	25.50 (19.00-51.50)	20.50 (7.75-43.00)	24.00* (14.25-40.25)	30.00 (18.00-38.00)	0.416	0.828
	A	23.00 (10.00-31.50)	28.00 (16.75-31.25)	31.50* (21.50-42.75)	26.50 (11.25-39.50)	30.00 (17.50-30.00)	30.00 (17.50-32.50)	**0.041**	
**PAF- AH in HPs (pmol/min/mg)**	T	265.64 (169.92-358.50)	198.20 (165.95-248.04)	217.11 (190.31-311.69)	209.26* (156.16-288.81)	188.57* (160.61-323.15)	190.99* (147.36-263.08)	**0.023**	0.396
	A	191.78 (157.18-275.14)	146.54 (137.00-221.96)	171.86 (130.92-297.72)	190.32 (164.64-229.98)	227.80 (179.40 - 270.80)	240.92 (166.19-254.90)	0.295	

Group_T: tenofovir-DF/emtricitabine/ efavirenz and Group_A: abacavir/lamivudine/ efavirenz. All the results are expressed as median values and interquartile range (25th-75th). HPs: Human Platelets

p_{time} displays the difference within the group during the overall 12-month treatment.

*: displays the significant difference of each time point with the baseline value ($p<0.05$).

P_{int} displays the difference between the two groups during the overall 12-month treatment.

$P_{specific-time-point}$ displays the significant difference between the two groups at a specific time point.
