# Supplementary material for: In vivo effect of two first-line ART regimens on inflammatory mediators in male HIV patients
Source: Lipids Health Dis. 2014 May 29;13:90. doi: 10.1186/1476-511X-13-90 (PMC4055908; doi:10.1186/1476-511X-13-90)
Supplement: Additional file 3 — Cytokines and VEGF levels in HIV-infected patients. [file 1476-511X-13-90-S3.pdf]

Additional File 3: Cytokines and VEGF levels in HIV-infected patients.

Cytokines (pg/mL)	Groups	Baseline (0 month)	1st month	3rd month	6th month	9th month	12th month	p_{time}	P_{int}
**IL-1β**	**T**	59.6 (42.4 - 75.8)	42.3 (26.5 - 72.8)	68.7 (48.5 - 87.1)	48.4 (40.0 - 69.2)	43.7 (32.1 - 54.8)	59.0 (38.1 - 78.6)	0.323	0.060
	**A**	71.7 (19.5 - 97.4)	64.1 (41.6 - 106.8)	88.4 (44.1 - 127.0)	40.6 (36.3 - 134.4)	39.6 (25.9 - 69.0)	53.3 (30.2 - 103.2)	0.276	
**IL-6**	**T**	44.5 (16.0 - 66.4)	76.6 (25.9 - 339.1)	33.6 (12.6 - 202.1)	34.1 (16.4 – 109.0)	44.5 (19.4 - 167.7)	86.9 (49.9 - 186.7)	0.505	0.173
	**A**	49.9 (23.5 - 153.3)	63.2 (10.4 - 170.8)	89.4 (14.6 - 260.0)	49.9 (20.2 - 91.1)	52.9 (12.4 - 115.6)	65.2 (43.0 - 165.5)	0.909	
**IL-10**	**T**	40.6 (14.9 - 135.5)	53.2 (20.8 - 89.4)	30.9 (16.0 - 101.9)	61.4 (25.1 - 140.4)	33.7 (13.2 - 76.3)	30.9 (18.9 - 105.9)	0.905	0.137
	**A**	22.0 (15.7 - 71.7)	92.1 (24.6 - 133.22)	52.7 (21.6 - 137.8)	31.1 (25.8 - 64.6)	33.7 (17.3 – 81.0)	38.5 (10.3 - 57.2)	0.478	
**TNFα**	**T**	206.1 (116.3 - 310.2)	154.41 (57.74 - 210.9)	221.7 (72.4 - 463.3)	121.1 (81.6 - 420.5)	90.3 (61.3 - 248.8)	77.6 (52.3 - 216.4)	0.539	0.136
	**A**	130.3 (71.2 - 281.7)	205.4 * (158.5 - 462.1)	202.6 (80.8 - 366.7)	128.5 (90.1 - 298.2)	91.4 (61.3 - 230.4)	95.3 (70.5 - 145.0)	0.183	
**IL-12p70**	**T**	53.1 (34.5 - 193.1)	111.0 (35.9 – 208.9)	62.8 (18.8 - 159.9)	87.7 (36.2 - 289.2)	38.0 (26.5 - 158.3)	67.1 (34.5 - 94.3)	0.778	0.104
	**A**	21.3 (5.0 – 217.0)	122.8 (42.1 - 243.2)	137.0 (38.2 - 217.3)	149.0 (26.7 - 259.5)	65.9 (41.1 - 150.0)	52.0 (18.77- 150.58)	0.483	
**VEGF**	**T**	5.9 (0.2 – 22.3)	13.5 (1.7 – 27.1)	5.3 (3.6 – 24.0)	8.9 (1.6 – 14.3)	11.7 (1.2 – 18.4)	9.7 (0.7 – 18.5)	0.991	0.200
	**A**	14.3 (2.3 – 28.0)	9.5 (3.1 – 12.0)	8.3 (0.2 – 17.7)	11.1 (6.3 – 15.6)	16.8 (5.1 – 27.8)	6.7 (1.0 – 12.0)	0.314	

Group_T: tenofovir-DF/emtricitabine/ efavirenz, Group_A: abacavir/lamivudine/ efavirenz. All the results are expressed as median values and interquartile range (25th-75th).

p_{time} and P_{int} display the difference within each groups or between the two groups during the overall 12-month treatment, respectively

*: displays the significant difference of each time point with the baseline value ($p<0.05$).
